# Case Report: Low-Frequency Repetitive Transcranial Magnetic Stimulation to Dorsolateral Prefrontal Cortex and Auditory Cortex in a Patient With Tinnitus and Depression

**DOI:** 10.3389/fpsyt.2022.847618

**Published:** 2022-03-09

**Authors:** Chun-Hung Chang, Wen-Lung Wang, Yu-Hui Shieh, Han-Yuan Peng, Chen-Syuan Ho, Hsin-Chi Tsai

**Affiliations:** ^1^An Nan Hospital, China Medical University, Tainan, Taiwan; ^2^Institute of Clinical Medical Science, China Medical University, Taichung, Taiwan; ^3^Department of Psychiatry and Brain Disease Research Center, China Medical University Hospital, Taichung, Taiwan; ^4^Department of Psychiatry, Tzu-Chi General Hospital, Hualien City, Taiwan; ^5^Institute of Medical Sciences, Tzu Chi University, Hualien City, Taiwan

**Keywords:** transcranial magnetic stimulation, tinnitus, depression, prefrontal cortex, low frequency

## Abstract

Repetitive transcranial magnetic stimulation (rTMS) has been widely used as a promising therapy for tinnitus. However, the exact target and stimulation sequence of rTMS that is most effective for treating tinnitus remains unclear. Here, we report a case of a 62-year-old man with treatment-refractory tinnitus and depression whose symptoms markedly improved after undergoing low-frequency rTMS over the right-side dorsolateral prefrontal cortex and left auditory cortex area. Our report indicates that low-frequency rTMS treatment that stimulates multiple brain regions sequentially is feasible and may clinically benefit patients with tinnitus and depression.

## Introduction

Tinnitus, a perception of sound in the absence of sound stimuli, has a prevalence of between 10 and 25% in the adult population (1, 2). Approximately 6 to 25% of patients with tinnitus experience a reduced quality of life because of tinnitus (3). The mechanisms of tinnitus are complex and heterogeneous, and they include the auditory system, somatosensory system, or a combination of these two systems (1). Numerous epidemiologic studies have revealed a strong association between tinnitus and depression (4). A systematic review of 28 studies (representing 15 countries and 9,979 patients with tinnitus) revealed a median prevalence of depression of 33% (with an interquartile range of between 19 and 49%) in patients with tinnitus (5). A brain imaging study reported the presence of shared neural networks in depression and tinnitus (6). However, current treatments are ineffective and unsatisfactory. Thus, the treatment response in patients with tinnitus and depression requires verification.

Repetitive transcranial magnetic stimulation (rTMS) is a novel non-invasive brain stimulation (NIBS) treatment that delivers a rapidly changing current through a coiled wire that is encased in plastic and placed above the scalp. According to Faraday's law of electromagnetic induction, the aforementioned method creates a magnetic field across the skull that subsequently generates an electric current in the targeted brain regions (7, 8). Studies have demonstrated the effectiveness of rTMS for neuropsychiatric disorders, and it is approved for treatment-resistant depression (9, 10). Moreover, reviews have suggested that NIBS treatments such as rTMS improve tinnitus (11, 12). In a pilot study (13) that enrolled 11 patients with tinnitus (eight of them had depressive symptoms), participants received their first cycle of rTMS to the primary auditory cortex for 5 days and their second cycle of rTMS to the primary auditory cortex and prefrontal cortex for 5 days; the researchers reported a significant improvement in Tinnitus Handicap Inventory (THI) (14) scores in patients with mild depression (10 ≤ BDI score < 16, *n* = 4) but not in patients with severe depression (BDI ≥ 16).

Herein, we report a case of a 62-year-old man with severe tinnitus and depression whose symptoms markedly improved after receiving low-frequency (LF) rTMS to the right-side dorsolateral prefrontal cortex and left auditory cortex.

## Case Report

The 62-year-old male patient presented to our brain stimulation center in 2021 and reported chronic tinnitus since 2014. According to the patient, he first perceived his tinnitus in both ears and near the left ear in his head, and it presented as a high-pitched ringing. He visited several ear, nose, and throat (ENT) clinics but was unsatisfied with their treatments. In April 2021, his tinnitus worsened. He visited the ENT outpatient clinic of a medical center for further evaluation. The results of brain imaging, electroencephalogram, and audiogram were within the normal range. The ENT physician noted that the patient exhibited depressive and anxious mood states associated with his refractory tinnitus. Therefore, he was referred to our psychiatric department for further evaluation.

The patient previously worked as the chief manager of a large company in 2014. His stress was attributed to his large workload, and tinnitus developed, followed by the first episode of depression with low mood, low energy, loss of interest, insomnia, and psychomotor retardation. Suicide ideation was also noted, but the patient denied making any suicide plans or attempts. His tinnitus worsened with depressive symptoms. Family history of depression and suicide attempts was noted. He denied having a history of bipolar disorder or engaging in alcohol or benzodiazepine abuse. A review of his medical history revealed a diagnosis of depressive disorder based on the Diagnostic and Statistical Manual of Mental Disorders, Fifth Edition (15). Prior medications included an adequate dose and duration of paroxetine (20 mg/day), duloxetine (30 mg/day) and then escitalopram (20 mg/day) with aripiprazole (5 mg/day) as augmentation treatment. His tinnitus and depression symptoms improved after 4 weeks of treatment. The patient's depressive episodes subsided, and his tinnitus became mild and intermittent. Medication use was discontinued after approximately 3 months of follow-up. However, in April 2021, a depressive episode occurred, and his tinnitus worsened. Depression with low mood, low energy, loss of interest, insomnia, psychomotor retardation and suicide ideation developed. He was brought to a psychiatric clinic, and 20 mg/d of escitalopram with aripiprazole (5 mg/day) was prescribed. However, the effects of the treatment were not satisfactory. Another antidepressant and psychotherapy for his depression were suggested. However, he refused antidepressants and had insufficient time for psychotherapy. Therefore, he was referred to our brain stimulation center for a repetitive transcranial magnetic stimulation (rTMS) consultation.

We used the THI to evaluate the severity of his tinnitus. The Beck Depression Inventory II (BDI-II) (16) and Hamilton Depression Rating Scale (HAM-D) (17) were used to evaluate the severity of his depression. The Beck Anxiety Inventory (BAI) was used to measure the severity of his anxiety symptoms (18). He scored 62, 40, 32, and 21 points on the THI, BDI-II, HAM-D, and BAI, respectively.

We measured the relevant scales before and after rTMS treatment. The patient provided signed informed consent prior to treatment. Each treatment session comprised rTMS applied to the right dorsolateral prefrontal cortex (R-DLPFC) and the left auditory cortex using an Apollo TMS Therapy stimulator (MAG & More, Germany) equipped with a figure-of-eight coil. The stimulation parameters of the LF rTMS that was administered were as follows: a 1-Hz 20-min train (1,200 pulses/session) at 110% of resting motor threshold was applied to the R-DLPFC followed by a 20-min train (1,200 pulses/session) that was applied to the left auditory area. For the dorsolateral prefrontal cortex (DLPFC) rTMS, the coils were held by stands and tangentially placed over the patient's DLPFC and rotated at a 45° from the midline. We performed coil localization in accordance with an algorithm developed by Beam et al. (19). Coils were placed over the Beam-F4 position when the R-DLPFC was being targeted, and they were placed halfway between T3 and T5 when the auditory cortex was being targeted (9). The sequential stimulation of the two regions (i.e., R-DLPFC followed by the left auditory cortex) was performed once per day for 5 days per week for 4 weeks for a total of 20 sessions.

After 2 weeks of therapy, the patient reported a considerable improvement in the severity of tinnitus, depression and anxiety. After 2 weeks, he scored 32, 18, 8, and 7 points on the THI, BDI-II, HAM-D, and BAI. After 4 weeks, he scored 9, 19, 8, and 6 points on the THI, BDI-II, HAM-D, and BAI. Suicidal thought disappeared after the treatment session. No clinically significant side effects were observed (Table 1).

**Table 1:** Clinical outcomes during the rTMS treatment.

**Symptoms**	**Baseline**	**2nd week**	**4th week**
THI	62	32	9
BDI-II	40	18	19
HAMD-17	32	8	8
BAI	21	7	6

## Discussion

To the best of our knowledge, this is the first reported case of the application of low frequency rTMS over two brain target regions in a patient with tinnitus and depression. Our patient exhibited significant clinical improvements after 20 treatment sessions without any noticeable side effects.

Park and his colleagues (13) enrolled 11 patients who presented with chronic tinnitus for more than 1 year; eight of these 11 patients had depressive symptoms (BDI score ≥ 10). These patients received their first rTMS to the primary auditory cortex for 5 days. After experiencing a tinnitus relapse (at between 1 and 6 months after the first treatment), they received their second rTMS to the primary auditory cortex and prefrontal cortex. Park et al. (13) discovered that the patients with mild depression (10 ≤ BDI score < 16, *n* = 4) exhibited significant improvement in their THI scores after receiving the second combined rTMS (ΔTHI score = 24.5).

However, in the aforementioned study, the patients with severe depression (BDI ≥ 16) did not exhibit a significant improvement in their THI scores. In the case of our patient, who had severe depression (BDI = 40, HAM-D = 32), a remarkable improvement was achieved using combined rTMS. Several reasons may contribute to this difference. First, the length and number of rTMS sessions can affect the response rate. Stimulation over a higher number of sessions may result in greater symptom reduction (9). Our patient underwent 20 treatment sessions (24000 R-DLPFC pulses and 24000 L-auditory cortex pulses), whereas the patients examined by Park et al. (13) only received 5 sessions of combined rTMS to two targets. Second, the DLPFC is more precise target than the prefrontal cortex for depression. We targeted the DLPFC by using an algorithm developed by Beam et al. (19). Coils were placed over the Beam-F4 position when the R-DLPFC was being targeted. Third, in the study conducted by Park et al., they first applied transcranial magnetic stimulation (TMS) for 5 days to the primary auditory cortex, but they did not apply combined rTMS to two brain targets (primary auditory cortex + prefrontal cortex) in the first TMS sessions. By contrast, we applied combined rTMS to two brain targets at the beginning of our treatment. Furthermore, for our stimulation, the two brain targets were the DLPFC followed by the auditory cortex. This sequence might have prompted the initial improvement in depression and tinnitus associated with mood states.

Studies have demonstrated that LF rTMS is a promising and well-tolerated therapeutic strategy for chronic tinnitus. A meta-analysis of 12 randomized controlled trials with 717 participants revealed that active rTMS was superior to sham rTMS in terms of its short- and long-term effects (6 months) on THI scores (12). In their meta-analysis, the overall short-term effect of rTMS on THI was calculated post-rTMS, and their results revealed that the mean difference (MD) of active rTMS was −7.05 (95% confidence interval [CI] = −11.65, −2.44), which was superior to sham rTMS (*P* < 0.003). The MD for the long-term effect (6 months) after rTMS was −7.01 (95% CI = −12.85, −1.18; *P* = 0.02). Furthermore, LF rTMS was demonstrated as a promising therapy for depression (9). A study reported that combined (multisite neuromodulation) protocols that target the prefrontal cortex and temporoparietal cortex simultaneously are effective in reducing the severity of tinnitus (20). A network meta-analysis examined 32 non-invasive stimulation interventions that included TMS and transcranial direct current stimulation (tDCS) (11). They revealed that the administration of cathodal tDCS to the left DLPFC combined with transcranial random noise stimulation to the bilateral auditory cortex was associated with the greatest improvement in tinnitus severity. rTMS with priming led to a greater reduction in tinnitus severity relative to that without priming. Therefore, on the basis of these studies and our study, clinicians may consider the application of LF TMS to multibrain areas either as a standalone treatment or for treatment augmentation in the management of tinnitus and depression.

Several physiopathogenic hypotheses may support the effectiveness of this combined stimulation. First, in other studies, hyperactivation and neural synchronization of the auditory cortex may have resulted in tinnitus (21, 22). LF TMS may modulate the neuronal activity and synaptic plasticity in the auditory cortex and non-auditory cortex, subsequently improving tinnitus (23, 24). Second, the improvement in tinnitus could be associated with the improvement in depression. We observed that between baseline and 2 weeks of stimulation, the improvement on the depressive level was greater (75% improvement in HAM-D scores) than on the tinnitus level (50% improvement). Depression may play a role in the development and aggravation of tinnitus (25). Antidepressants such as serotonin reuptake inhibitors improve not only depression but also tinnitus (26, 27). Image studies have revealed that the prefrontal cortex is involved both in the depression mechanism and in the development of tinnitus (28, 29). Therefore, LF rTMS over the right prefrontal cortex may improve depression-associated tinnitus. Third, 1-Hz rTMS treatment of the DLPFC could serve as priming for stimulation of the auditory cortex. Bilateral transcranial direct current stimulation (tDCS) targeting bilateral DLPFC has been shown to suppress tinnitus significantly in 30% of patients. Their results revealed that not only directly on the DLPFC but also indirectly on functionally connected brain areas relevant for tinnitus (30, 31). Therefore, the second stimulation targeting the auditory cortex of the tinnitus network can be enhanced by the priming stimulation over the prefrontal cortex.

However, our case report should be interpreted with caution because of the lack of a placebo control. This case report has several limitations. First, only one patient was evaluated. Further studies with larger samples and a randomized designed are required to verify the effects of the administration of LF rTMS to the R-DLPFC and auditory cortex on tinnitus and depression. Second, confounding factors (e.g., psychosocial stress or the COVID-19 pandemic) should be minimized by conducting event tracing during the treatment period.

## Conclusion

We reported that LF rTMS to the right DLPFC and left auditory cortex may improve severe tinnitus and depression. Future studies with larger sample sizes and randomized, double-blind, and placebo-controlled trials are warranted to verify our findings.

## Data Availability Statement

The original contributions presented in the study are included in the article/supplementary material, further inquiries can be directed to the corresponding author.

## Ethics Statement

Ethical review and approval was not required for the study on human participants in accordance with the local legislation and institutional requirements. The patients/participants provided their written informed consent to participate in this study.

## Author Contributions

C-HC and W-LW drafted the initial manuscript. Y-HS, H-YP, and C-SH provided expert opinions and reviewed the final submitted manuscript. H-CT critically reviewed the draft of manuscript and approved the final submitted version manuscript. All authors contributed to the article and approved the submitted version.

## Funding

This work was supported by grants from An Nan Hospital, China Medical University Hospital (ANHRF108-15 and ANHRF109-21).

## Conflict of Interest

The authors declare that the research was conducted in the absence of any commercial or financial relationships that could be construed as a potential conflict of interest.

## Publisher's Note

All claims expressed in this article are solely those of the authors and do not necessarily represent those of their affiliated organizations, or those of the publisher, the editors and the reviewers. Any product that may be evaluated in this article, or claim that may be made by its manufacturer, is not guaranteed or endorsed by the publisher.
